# Diagnostic Utility of the Prolactin Decrease Rate in the Diagnosis of Mild Hyperprolactinemia

**DOI:** 10.7759/cureus.71747

**Published:** 2024-10-17

**Authors:** Mutlu Güneş, Elif Güneş, Seher Çetinkaya Altuntaş

**Affiliations:** 1 Department of Endocrinology, Metabolism and Diabetes, Health Sciences University, Bursa Yuksek Ihtisas Training and Research Hospital, Bursa, TUR; 2 Department of Endocrinology, Metabolism and Diabetes, Health Sciences University, Bursa State Hospital, Bursa, TUR

**Keywords:** hyperprolactinemia, pool prolactin measurement, prolactin constant ratio, prolactin decrease rate, serial prolactin measurement, stress induced hyperprolactinemia

## Abstract

Background

Previous studies have yielded conflicting findings on the routine use of serial prolactin (PRL) measurement in patients with inconsistent signs or symptoms of mild hyperprolactinemia (HP). Therefore, we aimed to evaluate the effectiveness of serial PRL measurement and a previously undefined parameter which is the PRL decrease rate (PDR) method in the diagnosis of mild HP and prolactinoma.

Materials and methods

The data obtained from medical charts of patients in the sample population included serial PRL values at 0 minute (min), 30 min, and 60 min as well as macroprolactinemia (mPRL) levels. PDR was defined as the ratio of the difference between the PRL levels at 0 min and 60 min to the PRL level at 0 min.

Results

We obtained referral PRL (rPRL) levels from the files of 221 of 268 patients referred for HP. Of those referred, 153 (69.2%) had mild HP. Of the 165 patients in the serial PRL measurement group, HP was detected in 76 (46.1%), and stress-induced PRL elevation was found in 24 (14.5%). Of the 101 patients in the single PRL measurement group, HP was detected in 72 (69.9%; p<0.001). Moreover, a PDR score of 38.1% had 99% specificity and 26% sensitivity in HP exclusion, and a PDR score of 20.6% had 100% specificity and 30% sensitivity in prolactinoma exclusion.

Conclusion

In cases where clinical findings are insufficient, the serial PRL measurement method combined with the PDR for the diagnosis of prolactinoma and HP appears to be more reliable than the single measurement method.

## Introduction

Prolactin (PRL) is mainly synthesized in lactotroph cells in the pituitary and released in accordance with pulsatile and circadian rhythms [1]. PRL is under the control of stimulating and inhibitory factors. The factors that stimulate PRL release are suction, stress, and an increase in estrogen. These stimuli cause PRL to be secreted from the pituitary. Dopamine is the most important inhibitory factor. In the presence of sufficient dopamine in the pituitary portal system, PRL synthesis is inhibited by D2 receptors [2]. PRL is also secreted from the central nervous system, the immune system, the placenta, the breasts and the uterus [1]. When it is synthesized from the pituitary, PRL weighs 199 kilodaltons (kDa); then, it undergoes proteolysis and splits into units of 16 kDa and 23 kDa [1,3].

PRL combines with immune globulin (Ig) G antibodies to form a complex structure called macroprolactinemia (mPRL), mPRL causes a decrease in PRL clearance, leading to false positives PRL increase [4]. Studies have found that mPRL frequency is around 40% and that routine mPRL measurement prevents unnecessary examination and treatment [5,6]. However, guidelines do not recommend the routine use of mPRL measurement [7].

While some guidelines consider a single PRL measurement sufficient for the diagnosis of hyperprolactinemia (HP) [7], others recommend serial PRL measurement when inconsistencies are detected in clinical findings [8]. Previous studies have shown that clinical findings are unreliable in patients with mild HP [9,10]. Considering that the prevalence of pituitary microadenoma in the population is around 10% [9,11], in patients with mild HP who had inconsistent signs or symptoms a single PRL measurement followed by pituitary magnetic resonance (MR) sequencing is likely to increase overdiagnosis and treatment.

The main hypothesis of the present study was that; in patients with mild HP who had inconsistent signs or symptoms, the use of serial PRL measurement would lead to a more accurate decision-making process. We aimed to evaluate the effectiveness of serial PRL measurement and a previously undefined parameter which is the PRL decrease rate (PDR) obtained from this method in the diagnosis of mild HP and prolactinoma.

This article was previously posted to the ResearchGate preprint server in December 2022.

## Materials and methods

Inclusion criteria

The primary objective of this study is to evaluate the diagnostic utility of serial PRL measurements and the newly defined PDR in identifying mild HP and prolactinoma in patients with inconsistent clinical findings. The study population consisted of male and female patients aged 18 years old and over who were referred to the outpatient endocrinology clinic between January 2014 and April 2018.

Exclusion criteria

Patients who were previously diagnosed and under treatment; whose data could not be obtained from their files; who had a history of drugs or secondary diseases that would increase PRL, e.g. hypothyroidism, polycystic ovary syndrome (PCOS), chronic liver disease, chronic kidney failure; and who were pregnant were excluded from the analysis.

Study design and follow-up

A total of 268 patients referred for HP examination were identified. The venous puncture method was used to collect data on the following points: 0 minutes (min), 30 min and 60 min PRL levels; referral PRL (rPRL) and mPRL levels; creatinine (Cr), thyroid stimulating hormone (TSH), alanine transaminase (ALT) and aspartate transaminase (AST) values; height, weight and age statistics; and the presence or absence of oligomenorrhea, galactorrhea, hirsutism, headache and erectile dysfunction (ED). Pituitary magnetic resonance (MR) and drug use information had also been collected. This data was obtained from patient files and analyzed in the present study.

PRL levels were studied using the Abbott Architect Prolactin Reagent B7K76T Kit system. It covered the 90% range of expected and the entire expected normal range for men. The normal value range for men was 3.46-19.40 ng/ml, normal value range ​​for women was 5.18-26.53 ng/ml. Mild hyperprolactinemia is defined in men as a serum prolactin concentration between 19.50 and 100 ng/mL and in women 26.6 and 100 ng/mL [9]. In accordance with the guidelines, the serial PRL measurement method involved taking at least two or three measurements in 15-20 min intervals [8], and PRL values ​​that fell below normal limits at 60 min were considered indications of stress-induced HP. The PRL constant ratio (PCR) was defined as the ratio of the PRL level at 60 min to the PRL level at 0 min. PDR was defined as the ratio of the difference between the PRL levels at 0 min and 60 min to the 0 min PRL level.

Macroprolactinemia was measured in patients for whom an HP diagnosis could not be made based on clinical findings using the single or serial PRL measurement method. First, the basal PRL levels for mPRL were studied using the aforementioned kit. Then, after adding polyethylene glycol (PEG), the amount of PRL returned was studied, and the recovery rate was obtained. A monomeric PRL recovery rate of <40% compared to baseline was considered mPRL (+), a recovery rate of 40-60% was evaluated according to laboratory and clinical findings in accordance with the recommendation of the guidelines, and a recovery rate of >60% was considered true HP [8].

Ethical rules

The present study was approved by the ethics committee of the Health Sciences University, Bursa Yuksek Ihtisas Training and Research Hospital (2011-KAEK-25 2022/02-11). In light of the retrospective nature of the study, all procedures were performed as part of routine care and were conducted following the Strengthening the Reporting of Observational Studies in Epidemiology (STROBE) guidelines. The researchers affirm that they adhered to the Declaration of Helsinki. Informed consent was obtained from all individual participants included in the study.

Data availability

The data used for this manuscript will be made available upon reasonable request to the corresponding author.

Statistics

IBM® Statistical Package for the Social Sciences (SPSS) statistics 20 (IBM Corp., Armonk, USA) was used to compare the data. After the normal distribution was determined, an independent samples t-test was applied to the data with a normal distribution, and the Mann-Whitney U test was applied to compare the data that did not have a normal distribution. Pearson’s chi-squared test was used to compare ratios, and receiver operating characteristic (ROC) analysis was used to determine the best disease-related value. A p-value of <0.05 was considered statistically significant.

## Results

Clinical findings

We obtained rPRL levels from the files of 221 of 268 patients referred for HP. Of those referred, 153 (69.2%) had mild HP. The clinical signs and symptoms of the patient groups are shown in Table 1. Among the 129 patients with HP, 30 (23.3%) did not have galactorrhea or oligomenorrhea, 70 (54.3%) had oligomenorrhea or galactorrhea, and 29 (22.5%) had both conditions. Among the 21 patients with stress-induced HP, 10 (46.6%) had galactorrhea or oligomenorrhea, while none had both conditions.

**Table 1:** Distribution of symptoms and findings in HP and mPRL. HP: hyperprolactinemia, mPRL: macroprolactinemia, ED: erectile dysfunction

Variable	HP (+)	HP (-)	mPRL (+)	mPRL (-)
Female				
Oligomenorrhea, n (%)	84/129 (65.1%)	39/108 (36.1%)	13/35 (37.1%)	21/35 (60%)
Galactorrhea, n (%)	44/129 (34.1%)	20/108 (18.5%)	2/35 (5.7%)	16/35 (45.7%)
Hirsutism, n (%)	24/129 (18.6%)	27/108 (25%)	6/35 (17.1%)	8/35 (22.9%)
Headache, n (%)	6/129 (4.7%)	0/108 (0%)	0/35 (0%)	3/35 (8.6%)
Male				
ED, n (%)	14/18 (77.8%)	4/11 (36.4%)	3/7 (42.9%)	6/7 (85.7%)
Headache, n (%)	2/18 (11.1%)	0/11 (0%)	0/7 (0%)	0/7 (0%)

Macroprolactinemia

Macroprolactinemia measurement was requested for 85 of the patients suspected to have HP, and mPRL was detected in 43 (53.6%) of them. mPRL was measured in 39 patients in the single PRL measurement group and detected in 14 (35.9%) of them. mPRL was measured in 46 of the patients in the serial PRL measurement group and was detected in 29 (63.0%) of them (p=0.013). The demographic characteristics of these patients are shown in Table 2.

**Table 2:** Demographic and laboratory characteristics of patients HP and mPRL. Normally distributed data are presented as mean±standard deviation, while non-normally distributed data are presented as median (interquartile range). HP: hyperprolactinemia, mPRL: macroprolactinemia, F: female, M: male, BMI: body mass index, Cr: creatinine, TSH: thyroid stimulating hormone, ALT: alanin aminotransferase, AST: aspartate aminotransferase, PRL: prolactin, IQR: interquartile range.

Variable	HP (+)	HP (-)	p	mPRL (+)	mPRL (-)	p
Age (year)	39.5±10.8	35.3±8.8	0.003	36.7±10.2	37.7±10.3	>0.05
Gender (F/M)	129/19	109/11	>0.05	35/8	35/7	>0.05
BMI (kg/m^2^)	28.3±4.7	28.2±4.9	>0.05	28.4±4.3	27.1±4.3	>0.05
Cr (mg/dl)	0.7±0.19	0.7±0.17	>0.05	0.74±0.14	0.74±0.13	>0.05
TSH (μIU/mL)	2.1±1.2	2.2±1.2	>0.05	2.2±1.1	2.1±1.0	>0.05
AST (U/L)	20±7.1	18.6±6.4	>0.05	19.6±6.8	19.5±5.7	>0.05
ALT (U/L)	18.9±12.6	17.4±11.5	>0.05	17.6±13.8	16.5±7.3	>0.05
PRL(μg/L) 0 min	84 (IQR:86)	35 (IQR:37.3)	<0.001	55.5 (IQR:21.5)	59 (IQR:20)	>0.05
30 min	71 (IQR:58.5)	27 (IQR:28.8)	<0.001	51.5 (IQR:16.8)	56 (IQR:24)	>0.05
60 min	70 (IQR:56)	21.5 (IQR:29)	<0.001	51 (IQR:16)	53 (IQR:24)	>0.05

The etiological causes of HP

The demographic characteristics of the patients are shown in Table 2. HP was detected in 76 (46.1%) of the 165 patients in the serial measurement group and 72 (69.9%) of the 103 patients in the single measurement group (p<0.001). Of the 148 detected patients with HP, 1 had prolactinoma as a component of multiple endocrine neoplasia 1 (MEN1), and 1 had cosecretion of PRL with acromegaly. Meanwhile, radiological examination data were available for 91 of the 148 patients with HP. Microadenoma was detected in 16 patients without HP. In the 91 patients with HP for whom radiological examination data was available, microadenoma was detected in 68 (74.7%), macroadenoma was detected in 13 (14.3%) and no adenoma was detected in 10 (11.0%).

PRL levels, PDR, and PCR

The mean age of those with increased PRL due to stress was 33.3+8.0 years, while the mean age of those with HP was 38.6+10.2 years (p=0.017). Of the 165 patients in the serial measurement group, PRL levels were normal at 0 min in 36 (21.8%), HP was detected in 76 (46.1%), and a stress-induced increase in PRL was found in 24 (14.5%). In addition, mPRL was detected in 29, representing 17.6% of the total serial measurement group. Of the 103 patients in the single measurement group, HP was detected in 72 (69.9%), and normal PRL levels were found in 18 (17.3%). In addition, mPRL was detected in 14, representing 13.6% of the total single measurement group. There was a significant difference between the HP and mPRL detection rates using the two measurement methods (p<0.001 and p=0.028, respectively).

**Figure 1:**
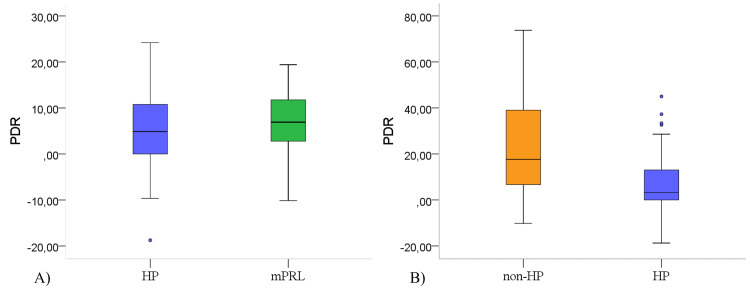
Comparison of the status of PDR in HP, non-HP and mPRL groups. (A) there was no difference in the decrease in the PRL level between HP and mPRL (p>0.05). The decrease in the PRL level was significantly less in HP (B) compared to non-HP (p<0.001). PRL: prolactin, PDR: PRL decrease rate, HP: hyperprolactinemia, mPRL: macroprolactinemia.

HP was excluded in 53.9% of the serial measurement group and 30.1% of the single measurement group. A PRL level below 36.50 at baseline (0 min) had 91% sensitivity and 42% specificity in HP exclusion. ROC curves of rPRL, the basal and PRL levels at 60 min for the diagnosis of prolactinoma and HP are shown in Figure 1. Regarding the diagnosis of prolactinoma, the area under the curve (AUC) values for rPRL, basal, and PRL levels at 60 min were 0.69, 0.71, and 0.75, respectively. Regarding the diagnosis of HP, the AUC values for rPRL, basal and PRL levels at 60 min were 0.66, 0.84 and 0.87, respectively. A PRL level at 60 min that was higher than 59 had 57% sensitivity and 91% specificity for detecting the presence of HP.

A PCR score of 79.4% or less had 100% sensitivity and 30% specificity for detecting the presence of prolactinoma. A PCR score of 88.2% or less had 100% sensitivity and 31% specificity for detecting the presence of macroadenoma. A PCR score of 61.9% had 99% sensitivity and 36% specificity for detecting the presence of HP.

**Figure 2:**
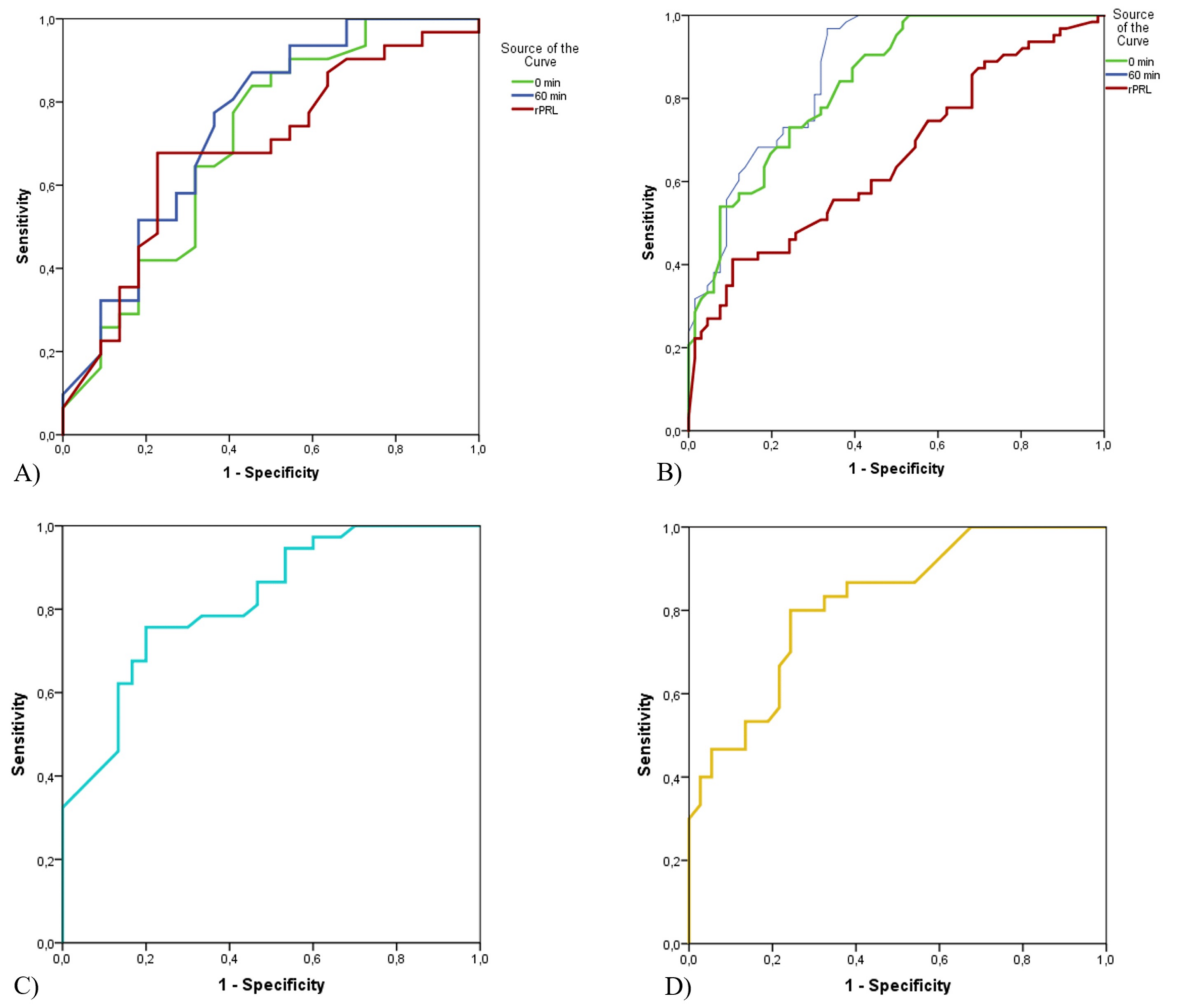
ROC curves of basal, 60 min and rPRL levels in the diagnosis of prolactinoma (A) and HP (B), ROC curves of PCR for the presence of prolactinoma (C); and ROC curves of PDR for the exclusion of prolactinoma (D) ROC: receiver operating characteristic curve, rPRL: referral prolactin,  HP:  hyperprolactinemia, PCR: prolactin constant ratio, PDR: prolactin decrease rate

Meanwhile, a PDR score that was greater than 38.1% had 99% specificity and 26% sensitivity for excluding HP. A PDR score of 20.6% or higher had 100% specificity and 30% sensitivity for excluding prolactinoma. The difference between the PDR levels of those with and without HP was significant, whereas the PDR levels of those with and without mPRL were similar (Figure 2).

Study population according to gender

Of the 146 female patients in the serial measurement group, 66 (45.2%) had HP, 36 (24.7) had normal PRL levels at 0 min, and 21 (14.4%) had stress-induced PRL elevation. In addition, in the serial measurement group, 23 of 37 female patients had mPRL positivity.

Of the 92 female patients in the single measurement group, 63 (68.5%) had HP and 17 (18.5%) had normal PRL levels. In addition, in the single measurement group 12 of 33 had mPRL positivity. While mPRL frequency was higher in the serial measurement group (p=0.031), more patients were diagnosed with HP in the single measurement group (p<0.001). Imaging data were available for 93 female patients with confirmed HP. Of those, microadenoma was detected in 76 (81.7%) and macroadenoma was detected in 8 (8.6%); no adenoma was detected 9 (9.7%).

HP was detected in 9 (52.6%) of the 19 male patients in the serial measurement group and 9 (81.8%) of the 11 male patients in the single measurement group. mPRL positivity was detected in six (66.7%) of the nine male patients in the serial measurement group and two (33.3%) of the six male patients in the single measurement group. Imaging data was available for 14 male patients with confirmed HP. Of those, macroadenoma was detected in five and microadenoma was detected in eight; no adenoma was detected in one of the fourteen patients.

## Discussion

A significant proportion of patients referred for HP consists of patients with mild HP. In patients with mild HP, the serial measurement method combined with the use of PCR and PDR for the diagnosis of prolactinoma and HP offers better management of the diagnostic process than the single measurement method. Furthermore, 14.5% of the patients in the serial measurement group in the present study had stress-induced PRL elevation. In contrast, the stress-induced HP percentages found in other studies were, 28.6% [9], 9% [10], 20% [12], and 24.2% [13].

It is important to consider whether the serial measurement time period used in a study could affect its results. For example, Whyte et al. [10] used a cannula and extended the measurement period to 120 min; as noted above, they observed a stress-induced HP rate that was lower than the one in the present study (9% vs 14.5%). However, there is no consensus on how long serial measurement should be extended [8]. Tsur et al. [13] found that PRL returned to normal at 60 min in 86 patients (24%) and at 90 min in 87 patients (24.2%); thus, prolonging the test period did not significantly contribute to the diagnostic process. Meanwhile, previous studies have stated that stress-induced PRL elevation stems from a fear of blood drawing [7, 14]. However, the stress-induced PRL elevation results in previous studies were similar to those of the present study, which examined blood samples taken using the venous puncture method, suggesting that PRL elevation may be independent of fear of venous puncture.

Previous studies have also shown that clinical findings are unreliable in patients with mild HP [9,10]. The most important complaint in the previous study and likewise in the present study HP populations was oligomenorrhea [10, 12]. In those with stress-induced HP, hirsutism and oligomenorrhea were detected at the same rate; however, oligomenorrhea and galactorrhea were not detected in any of the stress-induced HP patients. However, oligomenorrhea or galactorrhea were found in 36% and 14% of those without HP, respectively. In addition, Whyte et al. [10] found true HP in 17 (60%) of 28 asymptomatic patients. In the present study, HP was detected in 30 (23.3%) of the 129 patients without oligomenorrhea or galactorrhea. True HP can be detected in a significant portion of patients without a clinic, on the other hand, HP may not be detected in those patients with HP symptoms, which suggests that symptoms and clinical signs alone are not reliable in the management of patients with mild HP.

In the present study, PDR and PCR contributed to the diagnosis of HP and prolactinoma; such contributions have not been evaluated in previous studies. A PCR score of 79.4% or higher was present in all HP cases with microadenomas, and a PCR score of 88.2% or higher was present in all HP cases with macroadenomas. The present study’s findings indicated that PCR was better for demonstrating the presence of prolactinoma, while PDR was better for excluding prolactinoma. The ROC analysis revealed that measurements of rPRL, basal, and PRL levels at 60 min were similarly reliable in the diagnosis of prolactinoma. As a result, serial PRL measurement should be used instead of a single PRL measurement to determine the presence or absence of HP. In addition, since incidental pituitary adenomas are common in the normal population, PCR or PDR scores can guide the diagnosis and treatment of prolactinoma.

Although serial PRL measurement is typically performed in patients with mild HP and a second confirmation with mPRL is performed in cases of clinical uncertainty, the present study shows that these methods can be used together to exclude HP in a significant proportion of patients, regardless of clinical findings. Interestingly, the mPRL rate was higher in the serial measurement group than in the single measurement group, demonstrating the greater accuracy of the serial measurement method. However, similar findings or analyses were not found in the literature.

Although, as mentioned above, the guidelines recommend looking at symptoms and clinical findings first and then requesting mPRL in cases of laboratory and clinical inconsistencies [8], it is difficult to say that clinical findings alone are sufficient for the diagnosis of HP. In the serial measurement group, HP was excluded in 37 patients during the first stage (0 min: 22, 60 min: 15). In contrast, in the single measurement group, HP was initially excluded in 17 patients. In other words, the serial measurement method ultimately led to the exclusion of HP in 20 more patients than the other method (p<0.001). As such, the use of the serial measurement technique seems to support more accurate management of patients. Searle et al. and Francés et al. asserted that serial PRL measurement resulted in a reduction in unnecessary investigation and treatment costs [14, 15]. Conversely, Wilkinson et al. observed that the frequency of stress-induced HP was comparable between the single PRL measurement method and PRL sampling methods [16].

Regarding the limitations of the present study; using venous puncture in the serial PRL measurement method and performing it for 60 minutes may have prevented the detection of stress-induced HP in some patients. In addition, the retrospective nature of the study may have caused difficulties in obtaining patient data and the uneven distribution of the groups; therefore, some important findings may have been overlooked. Furthermore, the inability to establish a causality relationship in cross-sectional studies represents an additional limitation.

## Conclusions

The serial PRL measurement method combined with the use of PCR and PDR for the diagnosis of prolactinoma and HP appears to be more reliable than the single measurement method. Furthermore, serial PRL measurement combined with mPRL detection is the most accurate method of HP exclusion. Future prospective studies are warranted to validate the PDR's diagnostic accuracy across broader populations and to assess its implications for clinical management. While our results are encouraging, caution should be exercised in generalizing these findings without further validation.
